# Bridging the gap: the role of large language model refinement in readability in urology research

**DOI:** 10.1111/bju.16774

**Published:** 2025-05-19

**Authors:** Giovanni E. Cacciamani, Ethan Layne, Maria Giovanna Asmundo, Giorgio Ivan Russo

**Affiliations:** ^1^ USC Institute of Urology and Catherine and Joseph Aresty Department of Urology, Keck School of Medicine University of Southern California Los Angeles CA USA; ^2^ AI Center at USC Urology, USC Institute of Urology University of Southern California Los Angeles CA USA; ^3^ Keck School of Medicine University of Southern California Los Angeles CA USA; ^4^ Urology Section Humanitas Hospital Catania Italy; ^5^ Urology Section University of Catania Catania Italy

**Keywords:** large language model, artificial intelligence, natural language processing, readability, ChatGPT

**Abbreviations**
AI: artificial intelligence
ARI: Automated Readability Index
CLI: Coleman–Liau Index
FKG: Flesch–Kincaid Grade Level
FRE: Flesch–Kincaid Reading Ease
GFS: Gunning Fog Score
IQR: interquartile range
LASs: Layperson abstract and summaries
LLM: language model
OAs: original abstracts
SI: Smog Index
TRIPOD: Transparent Reporting of a multivariable prediction model for Individual Prognosis Or Diagnosis

The quality of scientific communication is crucial in ensuring that research findings are accessible and comprehensible to the intended audience. In fact, physicians and patients use internet very often in order to obtain and retrieve medical information [1, 2] and this highlight the importance of readability of research in order to maintain scientific accuracy.

An important aspect of scientific publishing is that an article's content should reflect several qualities, including readability, clarity, completeness, and accuracy. This is best achieved when the content is presented not just as a summary but as a more comprehensive piece of information, such as layperson abstracts and summaries. Interestingly, Pattenden et al. [3] analysed webpages from Google search dealing with ‘erectile dysfunction treatment’, with 91% containing specific treatment information and 21% being Health On the Net (HON) code certified. The median DISCERN score was low (35/80), and the median *Journal of American Medical Association* (JAMA) benchmark score was 1/4, indicating poor overall quality. Readability was at a high school level (mean score 12.32), above recommended levels for patient medical information.

Similarly, Gariscsak et al. [4] reported that according to the Flesch–Kincaid Reading Ease (FRE) scale, two‐thirds of articles retrieved in their analysis were classified as ‘difficult to read’, with nearly 30% categorised as ‘very difficult to read’. Readability scores showed a weak correlation with author count, word count, and reference count, while the authors’ country of origin had no significant impact. Based on these premises, our study analysed the readability, accuracy, completeness, and clarity of a set of urological research articles before and after a standard publication refinement process. The primary objective was to assess whether post‐publication processing improved readability while maintaining or enhancing the integrity of scientific reporting.

We selected all original articles and reviews published in the *BJU international* in 2024, retrieving a total of 258 articles. From these, we randomly selected 24 articles (two per month) that were analysed in the present research article. File S1 shows the list of retrieved references. The original abstracts (OAs) were individually inputted into the WebFX readability tool (https://www.webfx.com/tools/read‐able/) as previously done [5].

Layperson abstract and summaries (LASs) were then generated using a generative artificial intelligence (AI)‐based tool (www.pub2post.com) as previously reported [6]. We reported the FRE, Flesch–Kincaid Grade Level (FKG), Gunning Fog Score (GFS), Smog Index (SI), Coleman–Liau Index (CLI), and the Automated Readability Index (ARI). For FRE scores, a higher value corresponds with more readable text. For GFS, FKG, CLI, SMOG, and ARI, a lower value corresponds with more readable text [7]. We then employed a 5‐point Likert scale to grade accuracy, clarity and completeness of the outputs. Score that received ratings of 4–5 in all three criteria were considered appropriate. Conversely, if the outputs received ratings of 1–3 in any category, they were deemed inappropriate [7].

Median (interquartile range [IQR]) represent the continuous variables, while frequencies and percentages (%) represent the categorical variables. Non‐parametric was used to test mean differences. Spearman correlation test was used to test associations between variables. A two‐tailed test with a *P* < 0.05 was considered statistically significant. The statistical analysis was conducted using Statistical Package for the Social Sciences (SPSS), version 24.0 (IBM Corp., Armonk, NY, USA). Our analysis revealed significant improvements in readability of LASs compared to the OAs, as evidenced by substantial changes across all key readability indices. The FRE improved notably, with the median (IQR) score increasing from 24.25 (13.8–41.9) for OAs to 62.25 (57.05–69.95) for LASs, indicating a marked enhancement in text accessibility (*P* < 0.01). The median (IQR) FKG decreased from 14.2 (11.85–16.85) to 7.9 (6.9–9.0), reflecting a substantial simplification of sentence complexity and word structure (*P* < 0.01). The median (IQR) GFS, which measures the number of complex words per sentence, dropped from 16.9 (14.15–18.8) to 9.5 (7.7–10.5; *P* < 0.01). The median (IQR) SI, another complexity indicator, decreased from 12.45 (10.75–14.75) to 7.4 (6.4–8.15; *P* < 0.01). The median (IQR) CLI, which considers sentence length and syllable count, dropped from 16.3 (12.2–18.65) to 13.4 (11.95–14.5; *P* = 0.01). Finally, the median (IQR) ARI, another measure of text difficulty, showed a significant decrease from 13.15 (9.3–16.7) to 8.7 (7.45–9.6), reinforcing the general trend toward enhanced readability (*P* < 0.01). Figure 1 shows a summary of all results. A correlation analysis revealed a strong positive relationship between readability and clarity (*r* = 0.78, *P* < 0.01), suggesting that clearer writing styles contribute significantly to improved readability. No negative impact on accuracy or completeness was observed, reinforcing the notion that editorial refinement can improve accessibility without compromising scientific rigour.

**Fig. 1:**
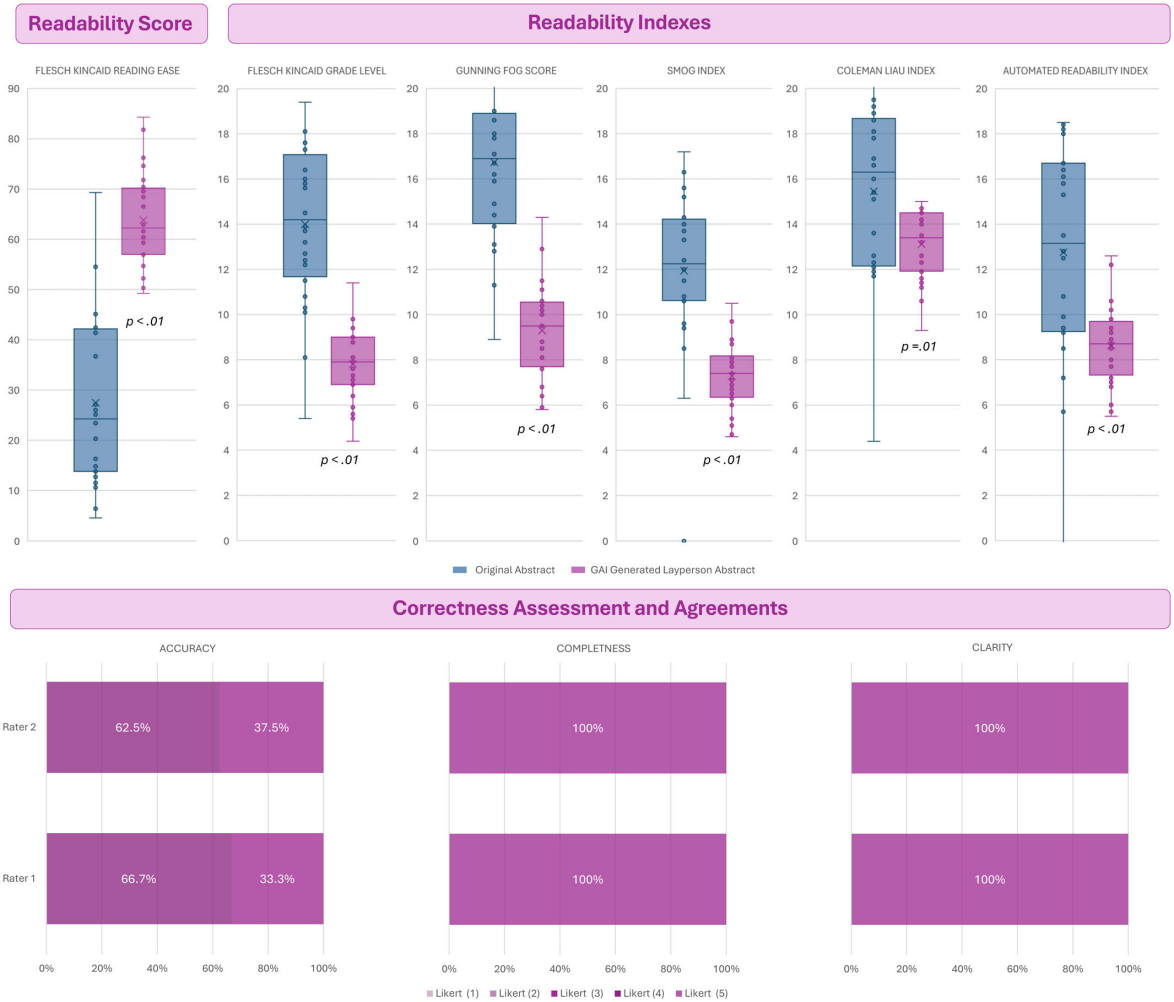
Summary of all results.

Inter‐rater evaluations showed that both reviewers consistently rated accuracy, completeness, and clarity between 4 and 5, with an overall agreement exceeding 90%. This consistency suggests that editorial interventions maintained the scientific integrity of the articles while improving readability.

The findings of this analysis support the hypothesis that editorial refinement substantially improves the readability of scientific articles without compromising accuracy, completeness, or clarity. The observed improvements in readability underscore the importance of structured writing, editorial interventions, and standardised formatting in scientific communication. Future research could explore the specific linguistic modifications that contribute most effectively to readability enhancement, as well as investigate whether improved readability correlates with greater citation rates or broader audience engagement. Additionally, refining readability without oversimplifying scientific content remains a crucial balance in academic publishing, necessitating continued methodological development in science communication.

Generative‐AI models are increasingly discussed and utilised in medical education [8] as well as creation of contents tailored for laypeople [5, 6]. Efforts are ongoing to assess how this technology can help healthcare providers deliver more readable and accessible patient education materials while ensuring that the information is derived from certified sources and maintains a high level of accuracy. Future directions should focus on implementing layperson abstracts and summaries alongside scientific summaries to ensure that no one is left behind. This approach would enable patients and their loved ones to access clear, reliable information about their disease, treatment options, expected outcomes, and ongoing clinical trials. One such initiative, Bridging Readable and Informative Dissemination with GenerativE Artificial Intelligence (BRIDGE‐AI; https://osf.io/8yz6d/), is currently ongoing in different medical disciplines, with results eagerly anticipated.

However, generative‐AI large language model (LLM) should be used with caution and humans should be always ‘in‐the‐loop’. In fact, the rapid advancement of LLMs challenges the traditional timelines of journal peer review and regulatory guidance, requiring faster adaptation. In response, researchers often rely on preprints and informal reporting methods. In this regard, Transparent Reporting of a multivariable prediction model for Individual Prognosis Or Diagnosis (TRIPOD)‐LLM provides a standardised reporting framework for LLMs in healthcare, expanding on the TRIPOD + AI guidelines. It addresses the specific challenges of biomedical applications through a structured checklist of 19 main items and 50 subitems, designed with a modular format to accommodate diverse research designs. Developed via an expedited Delphi process and expert consensus, TRIPOD‐LLM prioritises transparency, human oversight, and task‐specific performance reporting [9].

Given these findings, it is recommended that researchers and editors prioritise clarity and structured revisions in scientific manuscripts. Furthermore, the involvement of a layperson in order to assess the readability of scientific comment is something that in the future could be promising. Tools and techniques aimed at improving readability—such as linguistic simplification, logical organisation, and enhanced visual representation of complex data—can significantly aid in making research findings more accessible to diverse audiences, including clinicians, researchers, and policy‐makers.

## Disclosure of Interests

Giovanni E. Cacciamani: EditorAIpro (equity). The remaining authors have no disclosures.

## Disclaimer

ChatGPT was used for grammar correction of the present text. Giorgio Ivan Russo and Giovanni E. Cacciamani reviewed the contents and take full responsibility for the final output. A copy of the original drafting is stored.

## Supporting information


**File S1.** List of retrieved references.
