# Supplementary material for: Bridging the gap: the role of large language model refinement in readability in urology research
Source: BJU Int. 2025 May 19;136(3):356–8. doi: 10.1111/bju.16774 (PMC12343976; doi:10.1111/bju.16774)
Supplement: Supplementary file 1 — File S1. List of retrieved references. [file BJU-136-356-s001.docx]

Supplementary file 1. List of retrieved references.

(1–24)

1. Harding SL, Ilg MM, Bustin SA, Ralph DJ, Cellek S. Inhibition of phosphodiesterases 1 and 4 prevents myofibroblast transformation in Peyronie’s disease. *BJU Int* (2024) doi: 10.1111/bju.16631

2. Okhawere KE, Razdan S, Beksac AT, Saini I, Zuluaga L, Meilika K, Ucpinar B, Sheu R-D, Mehrazin R, Sfakianos J, et al. Novel bioabsorbable, low-dose rate brachytherapy device (CivaSheet®) with radical prostatectomy and adjuvant external beam radiation for the management of prostate cancer. *BJU Int* (2024) doi: 10.1111/bju.16617

3. Conduit C, Lewin J, Hong W, Sim I-W, Ahmad G, Leonard M, O’Haire S, Moody M, Hutchinson AD, Lawrentschuk N, et al. Pseudoephedrine for ejaculatory dysfunction after retroperitoneal lymph node dissection in testicular cancer. *BJU Int* (2024) 134:805–817. doi: 10.1111/bju.16481

4. Hayne D, Ong K, Swarbrick N, McCombie SP, Moe A, Hawks C, Viswambaram P, Conduit C, Liow E, Spalding L, et al. The SUB-urothelial DUrvalumab InjEction-1 (SUBDUE-1) trial: first-in-human trial in patients with bladder cancer. *BJU Int* (2024) 134:283–290. doi: 10.1111/bju.16325

5. Gabriel P-E, Pinar U, Lenfant L, Parra J, Vaessen C, Mozer P, Chartier-Kastler E, Rouprêt M, Seisen T. Comparative effectiveness of robot-assisted radical cystectomy with intracorporeal urinary diversion vs open radical cystectomy for bladder cancer. *BJU Int* (2025) 135:517–527. doi: 10.1111/bju.16565

6. Ben-David R, Lidagoster S, Geduldig J, Kolanukuduru KP, Elkun Y, Tillu N, Mandel A, Almoflihi M, Kaufmann B, Attalla K, et al. Undetectable pre-radical cystectomy circulating tumour DNA status predicts improved oncological outcomes. *BJU Int* (2025) 135:473–480. doi: 10.1111/bju.16556

7. Hahn AW, Manyam GC, Chapin BF, Zhang M, Yu Y, Pettaway CA, Chery L, Pisters LL, Ward JF, Gregg JR, et al. A phase II trial of apalutamide for intermediate-risk prostate cancer and molecular correlates. *BJU Int* (2024) 134:449–458. doi: 10.1111/bju.16414

8. Kapriniotis K, Loufopoulos I, Gresty HCM, Greenwell TJ, Ockrim JL. The utility of Martius fat pad in the repair of urogenital fistulae: review of current evidence. *BJU Int* (2024) 134:365–374. doi: 10.1111/bju.16350

9. García-Perdomo HA, Dávila-Raigoza AM, Summers E, Billingham L, Necchi A, Griffiths G, Spiess PE. Urethral cancer: a comprehensive review endorsed by the Global Society of Rare Genitourinary Tumours. *BJU Int* (2024) 134:175–184. doi: 10.1111/bju.16334

10. Vergamini LB, Ito W, Choi B N, Du HE, Sardiu ME, Neff D, Duchene DA, Molina WR, Whiles BB. Holmium:yttrium-aluminium-garnet laser with MOSES technology is more efficient than thulium fibre laser in supine mini-percutaneous nephrolithotomy. *BJU Int* (2024) 134:276–282. doi: 10.1111/bju.16392

11. Jussila I, Ahtiainen JP, Laakkonen EK, Siltari A, Kaipia A, Jokela T, Kärkkäinen M, Newton R, Raastad T, Huhtala H, et al. Transdermal oestradiol and exercise in androgen deprivation therapy (ESTRACISE): protocol. *BJU Int* (2024) 134:110–118. doi: 10.1111/bju.16361

12. Zhao Y, Chen G, Yushanjiang S, Zhao M, Yang H, Lu R, Qu R, Dai Y, Yang L. In vitro and in vivo study of antibacterial and anti-encrustation coating on ureteric stents. *BJU Int* (2024) 134:72–80. doi: 10.1111/bju.16326

13. Gerdtsson A, Negaard HFS, Almås B, Bergdahl AG, Cohn-Cedermark G, Glimelius I, Halvorsen D, Haugnes HS, Hedlund A, Hellström M, et al. Initial surveillance in men with marker negative clinical stage IIA non-seminomatous germ cell tumours. *BJU Int* (2024) 133:717–724. doi: 10.1111/bju.16289

14. Jeong HJ, Lee H, Choo MS, Cho SY, Jeong SJ, Oh S-J. Effect of detrusor underactivity on surgical outcomes of holmium laser enucleation of the prostate. *BJU Int* (2024) 133:770–777. doi: 10.1111/bju.16346

15. Vreeburg MTA, de Vries H-M, van der Noort V, Horenblas S, van Rhijn BWG, Hendricksen K, Graafland N, van der Poel HG, Brouwer OR. Penile cancer care in the Netherlands: increased incidence, centralisation, and improved survival. *BJU Int* (2024) 133:596–603. doi: 10.1111/bju.16306

16. Jefferson FA, Fadel A, Findlay BL, Robinson MO, Seyer AK, Koo K, Granberg CF, Boorjian SA, Anderson KT. The prevalence of impostor phenomenon and its association with burnout amongst urologists. *BJU Int* (2024) 133:579–586. doi: 10.1111/bju.16301

17. van Kessel CS, Palma CA, Solomon MJ, Leslie S, Jeffery N, Lee PJ, Austin KKS. Comparison of urological outcomes and quality of life after pelvic exenteration: partial vs radical cystectomy. *BJU Int* (2024) 133 Suppl 4:53–63. doi: 10.1111/bju.16299

18. McNicholas DP, Parr NJ. Image intensifier-guided transperineal prostate biopsy for patients without a rectum: novel technique. *BJU Int* (2024) 133:487–490. doi: 10.1111/bju.16279

19. Huang J, Chan SC, Pang WS, Liu X, Zhang L, Lucero-Prisno DE, Xu W, Zheng Z-J, Ng AC-F, Necchi A, et al. Incidence, risk factors, and temporal trends of penile cancer: a global population-based study. *BJU Int* (2024) 133:314–323. doi: 10.1111/bju.16224

20. Cleveland B, Norling B, Wang H, Gandhi V, Price CL, Borofsky M, Pais V, Dahm P. Tranexamic acid for percutaneous nephrolithotomy: an abridged Cochrane review. *BJU Int* (2024) 133:259–272. doi: 10.1111/bju.16244

21. Gordon P, Thompson D, Patel O, Ma R, Bolton D, Ischia J. Ureteric stenting outside of the operation theatre: challenges and opportunities. *BJU Int* (2025) 135:204–213. doi: 10.1111/bju.16533

22. den Hoedt S, van Veen FEE, Scheepe JR, Blok BFM. Bladder irrigation with tap water to reduce antibiotic use for urinary tract infections in catheter users. *BJU Int* (2025) 135:286–294. doi: 10.1111/bju.16552

23. Fang AM, Gregg JR, Pettaway C, Ma J, Szklaruk J, Bathala TK, Surasi DSS, Chapin BF. Whole-body MRI for staging prostate cancer: a narrative review. *BJU Int* (2025) 135:13–21. doi: 10.1111/bju.16514

24. Chiu PK, Liu AQ, Lau S, Teoh JY, Ho C, Yee C, Hou S, Chan C, Tang W, Bangma CH, et al. A 2‐year prospective evaluation of the Prostate Health Index in guiding biopsy decisions in a large cohort. *BJU Int* (2025) 135:71–77. doi: 10.1111/bju.16457
